# Temperature Change between Neighboring Days Contributes to Years of Life Lost per Death from Respiratory Disease: A Multicounty Analysis in Central China

**DOI:** 10.3390/ijerph19105871

**Published:** 2022-05-12

**Authors:** Chun-Liang Zhou, Ling-Shuang Lv, Dong-Hui Jin, Yi-Jun Xie, Wen-Jun Ma, Jian-Xiong Hu, Chun-E Wang, Yi-Qing Xu, Xing-E Zhang, Chan Lu

**Affiliations:** 1 Hunan Provincial Center for Disease Control and Prevention, Changsha 410005, China; hncdc_zcl@163.com (C.-L.Z.); hncdcmbkjdh@163.com (D.-H.J.); wangce0317@163.com (C.-E.W.); xu0510yiqing@163.com (Y.-Q.X.); yesgoshopping@163.com (X.-E.Z.); 2 Hunan Provincial Climate Center, Changsha 410007, China; xieyj_nju@163.com; 3 School of Medicine, Jinan University, Guangzhou 510632, China; mawj@gdiph.org.cn; 4 Guangdong Provincial Institute of Public Health, Guangzhou 511430, China; hzeros_hu@163.com; 5 XiangYa School of Public Health, Central South University, Changsha 410078, China

**Keywords:** respiratory diseases, ambient temperature, temperature change between neighboring days, years of life lost

## Abstract

Background: Many epidemiological studies have recently assessed respiratory mortality attributable to ambient temperatures. However, the associations between temperature change between neighboring days and years of life lost are insufficiently studied. Therefore, we assessed the attributable risk of temperature change between neighboring days on life loss due to respiratory disease. Methods: We obtained daily mortality and weather data and calculated crude rates of years of life lost for 70 counties in Hunan Province, Central China, from 2013 to 2017. A time-series design with distributed lag nonlinear model and multivariate meta-regression was used to pool the relationships between temperature change between neighboring days and rates of years of life lost. Then, we calculated the temperature change between neighboring days related to average life loss per death from respiratory disease. Results: The total respiratory disease death was 173,252 during the study period. The association between temperature change and years of life lost rates showed a w-shape. The life loss per death attributable to temperature change between neighboring days was 2.29 (95% CI: 0.46–4.11) years, out of which 1.16 (95% CI: 0.31–2.01) years were attributable to moderately high-temperature change between neighboring days, and 0.99 (95% CI: 0.19–1.79) years were attributable to moderately low-temperature change between neighboring days. The temperature change between neighboring days related to life loss per respiratory disease death for females (2.58 years, 95% CI: 0.22–4.93) and the younger group (2.97 years, 95% CI: −1.51–7.44) was higher than that for males (2.21 years, 95% CI: 0.26–4.16) and the elderly group (1.96 years, 95% CI: 0.85–3.08). An average of 1.79 (95% CI: 0.18–3.41) life loss per respiratory disease death was related to non-optimal ambient temperature. Conclusions: The results indicated that more attention should be given to temperature change, and more public health policies should be implemented to protect public health.

## 1. Introduction

Respiratory diseases are the third leading cause of death worldwide. According to the Global Burden of Disease (GBD) Study in 2019, chronic respiratory diseases caused 2.23 million deaths and 41.4 million years of life lost (YLL) [1]. Among these, chronic obstructive pulmonary disease (COPD) ranked first, with 1.18 million deaths and 32.8 million YLLs.

Numerous epidemiological researches have focused on the effects of absolute ambient temperature on respiratory disease mortality [2,3,4]. In the context of climate change, people may have a better adapt to the usual ambient temperature but less to temperature variability. However, the adverse impact of temperature variability on mortality is insufficiently studied [5]. Recently, many studies have reported that short-term variability of temperatures, such as temperature change between neighboring days (TCN) and diurnal temperature range (DTR), also play an important role in human health. Previous studies have mostly focused on DTR-related mortality [6,7,8]. However, DTR only considers the difference between daily maximum and minimum temperature. Recently, less evidence has assessed TCN exposure’s effects on mortality [9,10,11].

TCN considers the continuous process of unstable weather and has been implicated as an independent weather-related influencing factor for human health [12,13,14]. However, most of those studies concentrated on the number of death, which could not reveal the extent to which lives are shortened by exposure to non-optimal temperature changes. Compared with mortality, years of life lost (YLL) takes both death counts and age-at-death into consideration by allocating greater weight to deaths for younger populations [15,16,17]. A national study in China reported that the average life loss per death of respiratory mortality attributed to non-optimal temperature was 1.37 years [18]. One study found that the average life loss per death due to weight-temperature variability between neighboring days (a novel indicator integrated the effect of DTR and nocturnal temperature range) was 0.90 years [19]. All of those findings have important implications for understanding the impact of climate change on human health. However, none of the above-mentioned research explored the associations between TCN and YLL rates of respiratory diseases.

Therefore, we conducted a time-series analysis to examine the TCN-YLL rate association in Hunan Province, Central China. A distributed lag nonlinear model (DLNM) [20] was used to assess the impact of TCN on YLL across 70 counties. Then, we employed a multivariate meta-analysis to combine the effects of TCN. We also quantified the mortality burden attributed to TCN by calculating life loss per death.

## 2. Methods

### 2.1. Data Collection

**Figure 1:**
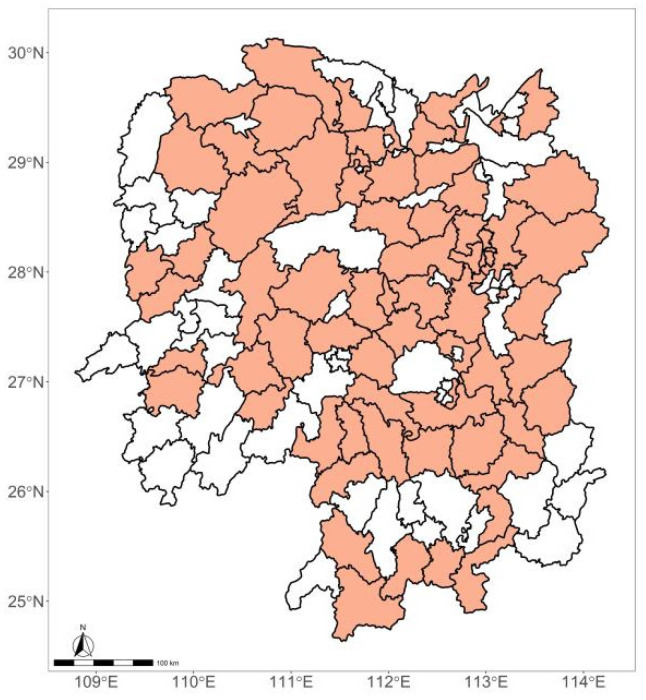
The geographical distribution of the 70 counties in Hunan Province, Central China.

In Hunan Province, temperature extremes exhibit a warming trend, and temperature variation has several significant warm-cold alternations and is more frequent than that in the whole of China [21,22]. To ensure enough statistical power, counties were selected if they met the inclusion criteria: a population size > 200,000 and/or an annual mortality rate > 4‰. We obtained daily counts of respiratory disease deaths and weather conditions in 70 counties from Hunan Province, Central China, from 1 January 2013 to 31 December 2017. The counties are displayed in Figure 1.

The causes of death were classified according to the International Classification of Diseases, 10th revision (ICD-10) [23]: J00-J98 for respiratory disease, J44 for chronic obstructive pulmonary disease (COPD) and J40-J47 for chronic lower respiratory tract infection (CLRTI). Only deaths from COPD and CLRTI were included because they were the most common causes of respiratory deaths. Daily mean temperature, minimum temperature (TMin), maximum temperature (TMax) and relative humidity (RH) of 698 meteorological monitoring stations across China were derived from the China Meteorological Data Sharing Service System. Then, we employed the Australian National University Splines (ANUSPLIN) to interpolate the temperature, TMin, TMax and RH at grid 0.01° × 0.01° resolution for all of China. Daily meteorological data from all 70 monitoring stations in Hunan Province, Central China, were extracted. The daily mean particulate matter (PM_10_) was obtained from the China National Environmental Monitoring Center. Since some counties were not included in the environmental monitoring system, we established a random forest model to estimate the mean PM_10_ for each county, as reported in previous research [19].

We obtained annual population data of 70 counties from 2013 to 2017 from the sixth national population census (http://www.stats.gov.cn, accessed on 23 July 2012). The daily total YLLs for each cause of respiratory death were calculated by summing the YLL for all respiratory deaths on the same day. We stratified the daily YLL by gender (male and female), age (0–74 years and ≥75 years), and the specific cause of death (CLRTI and COPD). Then, we computed the rate of daily YLL (YLL per 100,000 population per year) by dividing the daily YLL by the corresponding population size.

According to published literature [24], we used a composite index of intraday and interday temperature variability by estimating the standard deviation of TMax and TMin between two neighboring days. The TCN was calculated as follows.

TCN = SD (TMinlag0, TMaxlag0, TMinlag1, TMaxlag1)

### 2.2. Statistical Analysis

A two-stage analytic approach explored the relationships between TCN and the YLL rate. We used a time series model to derive estimates of county-specific exposure-response associations in the first stage. We applied a multivariate meta-analysis model to pool the estimated county-specific overall cumulative TCN-YLL rate associations in the second stage.

**First stage of analysis**. We used a DLNM linked with a Gaussian distribution function to model the relationships of the TCN-YLL rate in each location. The DLNM was adjusted for some potential confounders, such as time trends, an indicator of the day of the week (DOW), RH and PM_10_. The DLNM model was described as follows.

$$E\left(Y_{t}\right)=\alpha +cb\left(TCN_{t},lag\right)+cb\left(Tem_{t},lag\right)+ns\left(time_{t},df*t\right)+ns\left(RH_{t},df\right)+\beta_{1}DOW_{t}+\beta_{2}PM_{10t}$$

A cross-basis function (*cb*) was applied to model the complex nonlinear and lagged effects associations of TCN and temperature (Tem) with the YLL rate: nonlinear association modeled by a quadratic B-spline with three knots (10th, 50th and 90th); delayed effect modeled by a natural cubic B-spline with a maximum lag period of 21 days [25,26]. The temperature was adjusted as a potential confounder when we explored the association between TCN and YLL rate. A natural cubic spline (*ns*) was used to control time trends and the effect of RH, with 7 degrees of freedom (*df*) per year for time trends and 3 *df* for RH. A dummy variable was introduced to control the confounding effect of DOW.

**Second stage of analysis**. A multivariate meta-analysis was used to pool the location-specific cumulative exposure-response associations obtained from the first stage. Because the combined curves of TCN and YLL rates were w-shaped, we defined the two lowest points of the w-shaped curve as the TCN corresponding to the minimum YLL rate (MYTc1) and MYTc2. The corresponding minimum YLL percentile of the TCN was defined as MYPc1 and MYPc2, respectively. We divided TCN into four groups (low, moderately low, moderately high and high) by quantiles (≤MYTc1, MYTc1 to the median, the median to MYTc2, and >MYTc2). The temperature corresponding to the minimum YLL rate (MYT) was treated as the centering value of the association between ambient temperature and the YLL rate. Therefore, we divided ambient temperatures into four groups: extreme cold, moderate cold, moderate heat, and extreme heat by quantiles (≤2.5th centile, 2.5th centile to MYT, MYT to 97.5th centile and >97.5th centile, respectively). We calculated the attributable YLL stratified by sex (male, female), age group (0–75 years old, ≥75 years old) and cause of death (CLRTI, COPD).

To quantify the mortality burden of non-optimal TCNs and their components, we further calculated the mean years of life lost per death from respiratory disease attributable to the TCN. The YLL of respiratory diseases attributable to non-optimal TCNs can be calculated by population and YLL rates. Then, the daily attributable YLL of respiratory diseases at each location was added to obtain the total TCN-attributable YLL. We calculated the average YLL per case resulting from TCN by dividing the sum of attributable YLL by the number of cases in the different subgroups. The non-optimal ambient temperature mortality burden was estimated using the same methods mentioned above.

**Sensitivity analyses**. Sensitivity analyses were performed to test the robustness of our results. We employed the *df* of time from 6 to 9 years, modified maximum lag periods of 7, 14, and 21 days, explored the exposure-response relationship with and without PM_10_, and estimated the exposure-response relationship with and without RH to fit the models.

All data analyses were performed using R software (version 4.0.0). The “dlnm” [20] and “mvmeta” [27] packages were mainly used. A two-tailed *p* values < 0.05 was considered to be statistical significance.

## 3. Results

### 3.1. Descriptive Statistics

Table 1 shows the distribution of average daily YLL rates of respiratory diseases and weather conditions in 70 locations within the study period. The total respiratory disease deaths were 173,252. The mean daily YLL rates for respiratory diseases, CLRTI and COPD were 2.3/100,000, 1.8/100,000 and 0.6/100,000, respectively. The average daily YLL rates for males (2.8/100,000) and the elderly population (age 75+ years, 26.6/100,000) had much higher YLL rates than those for females (1.8/100,000) and the younger population (age 0–74 years, 1.3/100,000), respectively. We observed large variations in TCN ranging from 0.4 to 13.0, with a mean of 4.6.

### 3.2. TCN-YLL Rates Associations

**Figure 2:**
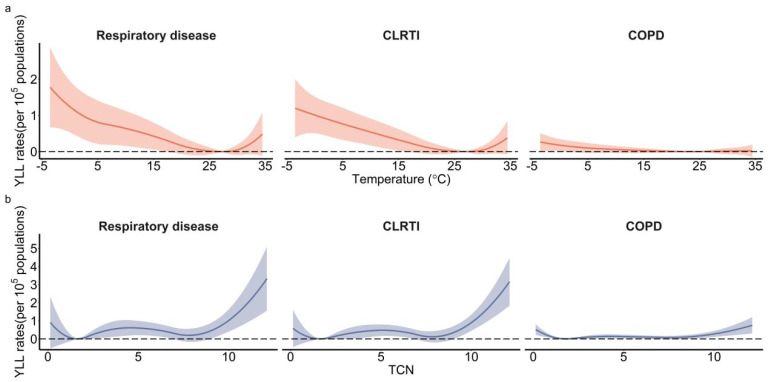
The cumulative exposure-response curves between temperature/TCN and YLL rates for total respiratory disease and cause-specific respiratory disease from 70 counties in Hunan Province, Central China, 2013−2017. (**a**): ambient temperature and YLL rate; (**b**): TCN and YLL rate. TCN: temperature change between neighboring days; CLRTI: chronic lower respiratory tract infection; COPD: chronic obstructive pulmonary disease.

Inverted *J* shapes were observed between daily mean temperature and YLL rates of overall and cause-specific respiratory diseases. The MYT for respiratory diseases, CLRTI and COPD were 27.23 °C, 26.87 °C and 23.66 °C, respectively. The cold temperature had a much larger impact on the YLL rates than the hot temperature (Figure 2a).

**Figure 3:**
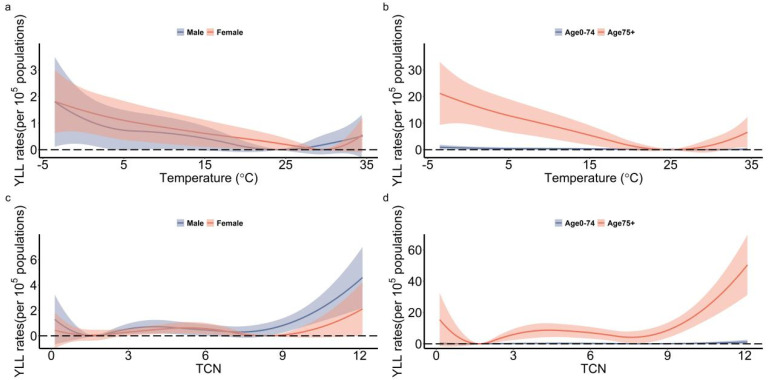
The exposure-response curves between temperature/TCN and YLL rates for different sub-group of sex and age in 70 counties from Hunan Province, Central China, 2013−2017. (**a**,**b**): ambient temperature and YLL rate; (**c**,**d**): TCN and YLL rate. TCN: temperature change between neighboring days.

W-shapes were observed between TCN and YLL rates of respiratory diseases and cause-specific respiratory diseases. The MYTc1 for respiratory diseases, CLRTI and COPD were 1.63, 1.64 and 1.84, respectively. The MYTc2 for respiratory diseases, CLRTI and COPD were 7.79, 7.82 and 7.49, respectively. High TCN had a much larger impact on the YLL rates than low TCN (Figure 2b). In the subgroup analysis, non-optimal TCN had a much greater impact on YLL rates in males than females, whereas non-optimal ambient temperature had a much greater impact on YLL rates in females than males. Both non-optimal TCN and ambient temperature had a much greater impact on YLL rates in the elderly than in the younger population (Figure 3).

### 3.3. Mortality Burden Attributable to TCN

The mean life loss per respiratory disease death attributed to non-optimal ambient temperature was 1.79 (95% CI: 0.18–3.41) years, of which 1.60 (95% CI: 0.17–3.04) years were associated with moderately cold temperature. In the subgroup analyses, females (3.43 years, 95% CI: 0.69–6.16) and the younger group (3.53 years, 95% CI: −1.77–8.82) had higher life loss per respiratory disease death than males (1.48 years, 95% CI: −0.22–3.16) and the elderly group (1.49 years, 95% CI: 0.55–2.43). For cause-specific respiratory diseases, the life loss associated with temperature exposures was 1.84 years (95% CI: 0.35–3.43) and 0.62 years (95% CI: −0.67–1.91) for CLRTI and COPD, respectively (Table 2).

Nonoptimal TCN contributed to more life loss per death from respiratory diseases than ambient temperature. The mean life loss per respiratory disease death attributed to TCN was 2.29 (95% CI: 0.46–4.11) years, of which 1.16 (95% CI: 0.31–2.01) years were associated with moderately high TCN. In the subgroup analyses, females (2.58 years, 95% CI: 0.22–4.93) and the younger group (2.97 years, 95% CI: −1.51–7.44) had higher life loss per respiratory disease death than males (2.21 years, 95% CI: 0.26–4.16) and the elderly group (1.96 years, 95% CI: 0.85–3.08). For cause-specific respiratory diseases, the life loss associated with TCN exposures was 2.11 years (95% CI: 0.31–3.90) and 1.95 years (95% CI: 0.25–3.65) for CLRTI and COPD, respectively (Table 3).

### 3.4. Sensitivity Analysis

The results did not show a difference in estimated effects by changing the *df* for the time from 6/year to 9/year, extending the lag period of the TCN effect (7, 14 and 21 days), or adjusting the model with and without PM_10_ or RH (Supplementary Figure S1).

## 4. Summary and Discussion

We conducted a multicenter study in Hunan Province, Central China, to estimate the associations between TCNs and life loss per death from respiratory diseases. Our findings showed that a mean of 2.29 years of life loss per death from respiratory diseases was attributed to non-optimal TCNs. Most of the years of life loss were attributed to moderate TCNs since moderately TCN has a wider range than other TCN components, and days with moderately high TCN are more frequent than high TCN. To our knowledge, this is the first DLNM-based multicenter study to explore the effects of TCN on life loss per death from respiratory disease.

Previous studies have reported the relationship between ambient temperature or temperature variability and mortality or YLL [8,28,29]. A large multicountry analysis found that temperature variability was linked to an increased risk of death, even after controlling for the effects of daily mean temperature [5]. Our study showed that ambient temperature is responsible for a substantial fraction of life loss. A mean of 1.79 years of life lost per death was attributed to non-optimal ambient temperature. These findings provided evidence that ambient temperatures are linked to increases in daily mortality, but they did not consider the associations between TCN and life loss per death from disease.

In line with the findings of previous studies [10,30], we found that a non-optimal TCN markedly elevated the life loss from respiratory disease. Those studies reminded us that the government, researchers and public should give more attention to sudden temperature changes. The adverse effects of non-optimal temperature change are biologically plausible because the human thermoregulatory system cannot respond quickly and efficiently to sudden changes in temperature [31]. People may feel uncomfortable with sudden changes in temperature because they are not well prepared for temperature variability, not only physiologically but also behaviorally [32,33]. Rapid temperature variability is associated with dehydration, salt depletion and increased surface blood circulation, which may cause TCN-related life loss [34]. More measures should be taken to raise people’s awareness of TCN-respiratory relationships. Two major cause-specific respiratory diseases, CLRTI and COPD, contributed to a large proportion of the TCN-related respiratory YLL rate. Therefore, people with CLRTI and COPD should reduce the time for outdoor activities and pay more attention to the indoor temperature on days with large temperature changes.

Considering the demographic characteristics of this study, we add to evidence that TCNs have a great impact on life loss for different groups. In this study, we found that females were more vulnerable to rapid changes in temperature, which is in accord with previous studies [35,36]. However, prior epidemiologic studies have also reported inconsistent findings. Some studies estimated the temperature-related life loss and found that males were more vulnerable to non-optimal temperature exposure [18,24]. This difference indicated that the effect of gender differences on the association of TCN-YLL rate might change with population and region. A substantial number of studies have shown that elderly individuals are more susceptible to unfavorable temperatures. Zhang et al. found that the elderly (≥65 years) had a higher respiratory death risk attributable to temperature than the young (0–64 years) [37]. However, we observed more life loss per death for the younger population. This discrepancy indicated that the respiratory death burden attributable to non-optimal TCNs was heavier for young death, even if the absolute count of respiratory deaths was smaller than that of the elderly. Individuals who die at a younger age would suffer from more YLL than elderly, which could explain the discrepancy we found.

Some limitations must be acknowledged in this study. First, for the ecological study, we used environmental monitoring data that replaced individuals’ accurate exposure temperature to investigate the effect of TCN, which could introduce some exposure measurement errors. Second, the data of this study is from 70 counties in Hunan Province, Central China, so the results cannot be generalized to populations in other areas. In order to explore the influence of weather patterns, the methodologies could be extended to populations in other study areas. Third, this research did not consider the direction of the temperature change. Fourth, we did not control other confounding factors that might affect the relationship between TCN and life loss per death, such as air pollutants other than PM_10_, health service conditions and living conditions.

## 5. Conclusions

We found that TCN is an independent risk factor for life loss of respiratory diseases. Females and the younger group were particularly susceptible to TCN. Our study highlights that targeted intervention strategies are necessary to reduce the TCN-related life loss of respiratory diseases.

## Figures and Tables

**Table 1:** Characteristics of study variables in 70 counties in Hunan Province, Central China, 2013–2017.

Characteristics	Mean	SD	Min	P_25_	P_50_	P_75_	Max
Daily respiratory YLL rate	2.3	3.2	0	0	1.4	3.4	65.5
Sex							
male	2.8	4.7	0	0	0	4.1	105.6
female	1.8	3.8	0	0	0	2.6	104.6
Age							
0–74	1.3	2.7	0	0	0	1.8	62.7
≥75	26.6	38.7	0	0	10.8	42.3	1176.3
Cause-specific							
CLRTI	1.8	2.5	0	0	0.9	2.7	65.5
COPD	0.6	1.4	0	0	0	0	24.2
Meteorological variable							
Daily mean temperature (°C)	17.7	8.3	−3.5	10.4	18.5	24.7	34.5
TCN	4.6	2.0	0.4	3.1	4.5	5.8	13.0
Relative humidity (%)	77.8	11.0	30.7	70.5	78.9	86.1	100
Wind speed	1.9	1.2	0	1.1	1.6	2.4	17.9
Pressure	995.2	20.7	859.4	988.5	997.6	1006.8	1041.3
PM_10_ (μg/m^3^)	85.5	44.7	8.3	54.3	75.4	107.8	530.6

YLL: years of life lost; CLRTI: chronic lower respiratory tract infection; COPD: chronic obstructive pulmonary disease; TCN: temperature change between neighboring days.

**Table 2:** Non-optimal ambient temperatures contribute to life loss per death from respiratory diseases in 70 counties in Hunan Province, Central China, 2013–2017.

Type	MYT	MYP	Total	Extreme Cold	Moderate Cold	Moderate Heat	Extreme Heat
Overall	27.23	85.7	1.79 (0.18–3.41)	0.15 (0.06–0.24)	1.60 (0.17–3.04)	0.01 (−0.04–0.07)	0.02 (−0.01–0.06)
Sex							
Male	24.15	72.4	1.48 (−0.22–3.16)	0.12 (0.02–0.22)	1.24 (−0.09–2.58)	0.09(−0.12–0.30)	0.04 (−0.02–0.09)
Female	29.50	93.6	3.43 (0.69–6.16)	0.23 (0.09–0.36)	3.17 (0.61–5.73)	0 (−0.01–0.01)	0.02 (−0.01–0.06)
Age							
0–74	29.37	93.2	3.53 (−1.77–8.82)	0.24 (−0.02–0.49)	3.27 (−1.68–8.21)	0 (−0.02–0.03)	0.02 (−0.05–0.09)
≥75	24.95	76.0	1.49 (0.55–2.43)	0.13 (0.07–0.18)	1.29 (0.52–2.06)	0.04 (−0.05–0.13)	0.03 (0–0.05)
Cause-specific							
CLRTI	26.87	84.3	1.84 (0.35–3.43)	0.15 (0.08–0.23)	1.65 (0.33–2.98)	0.02 (−0.04–0.08)	0.02 (−0.01–0.06)
COPD	23.66	70.1	0.62 (−0.67–1.91)	0.08 (0–0.15)	0.51 (−0.43–1.45)	0.03 (−0.19–0.25)	0.01 (−0.04–0.06)

MYT: minimum YLL temperature; MYP: minimum YLL percentile of the daily temperature; CLRTI: Chronic lower respiratory tract infection; COPD: chronic obstructive pulmonary disease.

**Table 3:** Nonoptimal TCN contributes to life loss per death of respiratory diseases in 70 counties in Hunan Province, Central China, 2013–2017.

Type	MYTc1	MYPc1	MYTc2	MYPc2	Total	Low TCN	Moderately Low TCN	Moderately High TCN	High TCN
Overall	1.63	4.4	7.79	94.9	2.29 (0.46–4.11)	0.02 (−0.03–0.07)	0.99 (0.19–1.79)	1.16 (0.31–2.01)	0.11 (−0.01–0.24)
Sex									
Male	1.64	4.5	7.34	92.4	2.21 (0.26–4.16)	0.03 (−0.04–0.09)	1.06 (0.23–1.88)	0.93 (0.05–1.82)	0.20 (0.02–0.38)
Female	1.56	3.9	8.37	97.1	2.58 (0.22–4.93)	0.02 (−0.10–0.14)	1.01 (−0.02–2.03)	1.52 (0.36–2.68)	0.03 (−0.01–0.08)
Age									
0–74	1.45	3.0	8.06	96.0	2.97 (−1.51–7.44)	0.01 (−0.08–0.09)	1.36 (−0.72–3.43)	1.53 (−0.54–3.6)	0.07 (−0.18–0.31)
≥75	1.66	4.7	7.59	93.9	1.96 (0.85–3.08)	0.02 (−0.01–0.06)	0.84 (0.37–1.32)	0.96 (0.45–1.47)	0.14 (0.05–0.23)
Cause-specific									
CLRTI	1.64	4.5	7.82	95.0	2.11 (0.31–3.90)	0.02 (−0.03–0.06)	0.83 (0.06–1.60)	1.15 (0.29–2.02)	0.11 (−0.01–0.22)
COPD	1.84	6.3	7.49	93.3	1.95 (0.25–3.65)	0.07 (0.02–0.11)	0.83 (0.11–1.55)	0.90 (0.11–1.69)	0.15 (0.01–0.30)

TCN: temperature change between neighboring days; MYTc: minimum YLL rate TCN; MYPc: minimum YLL percentile of the TCN; CLRTI: chronic lower respiratory tract infection; COPD: chronic obstructive pulmonary disease.

## Supplementary Materials

The following supporting information can be downloaded at: https://www.mdpi.com/article/10.3390/ijerph19105871/s1. Figure S1: Sensitivity analyses on the impact of df(/year), lag, PM_10_ and RH for the pooled exposure-response curves between TCN and YLL rate.
