# Supplementary material for: Temperature Change between Neighboring Days Contributes to Years of Life Lost per Death from Respiratory Disease: A Multicounty Analysis in Central China
Source: Int J Environ Res Public Health. 2022 May 12;19(10):5871. doi: 10.3390/ijerph19105871 (PMC9141323; doi:10.3390/ijerph19105871)
Supplement: Supplementary file 1 [file ijerph-19-05871-s001.zip › ijerph-1657961-supplementary.pdf]

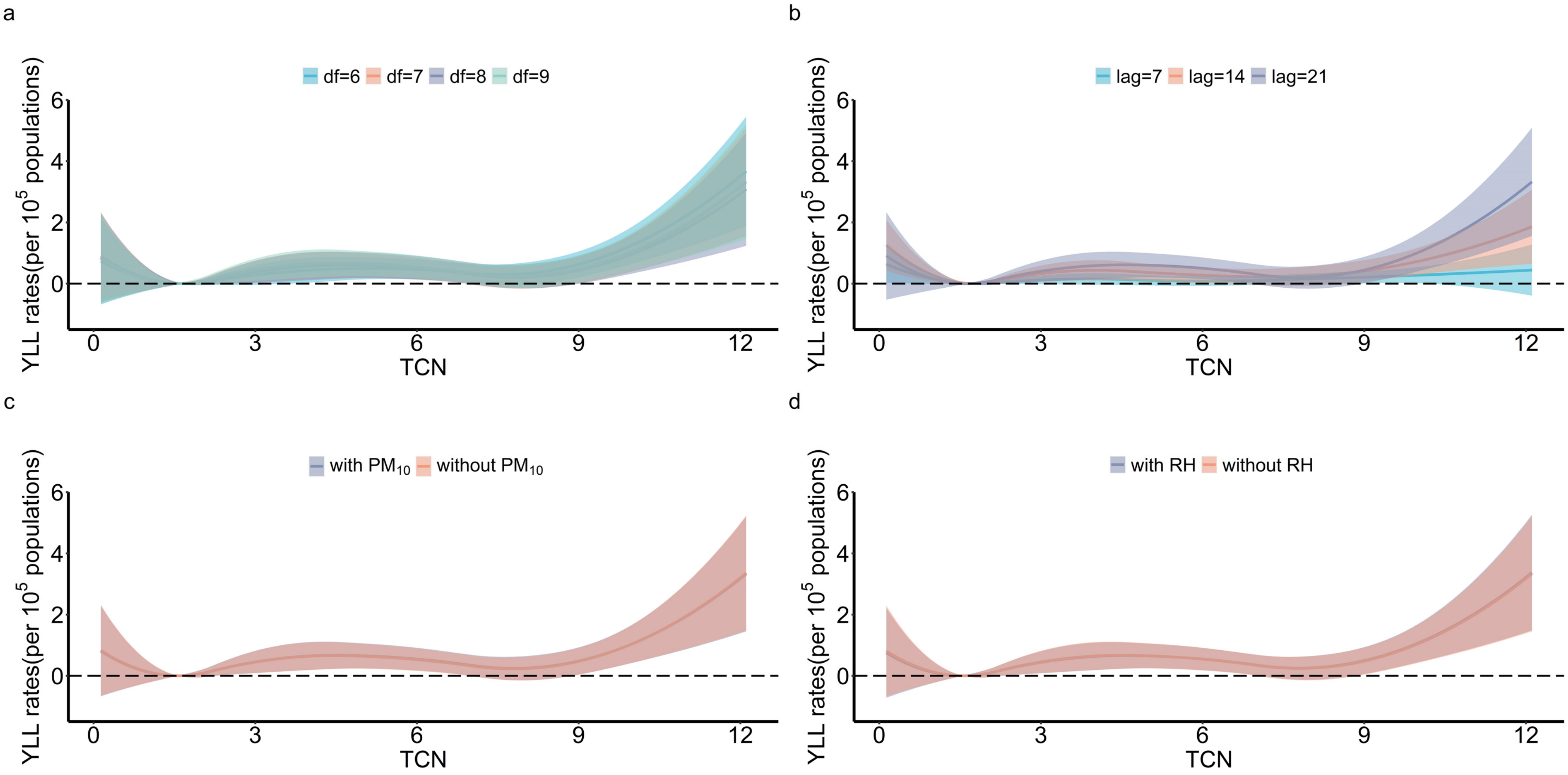

Figure S1: Sensitivity analyses on the impact of df(/year), lag, PM10 and RH for the pooled expo-sure-response curves between TCN and YLL rate.
